# Influence of the prone position on a stretcher for pregnant women on maternal and fetal hemodynamic parameters and comfort in pregnancy

**DOI:** 10.6061/clinics/2017(06)01

**Published:** 2017-06

**Authors:** Claudia Oliveira, Marco Antonio Borges Lopes, Agatha Sacramento Rodrigues, Marcelo Zugaib, Rossana Pulcineli Vieira Francisco

**Affiliations:** I Fisioterapia, Universidade Santa Cecilia, Santos, SP, BR; II Departamento de Obstetricia e Ginecologia, Faculdade de Medicina da Universidade de Sao Paulo, Sao Paulo, SP, BR; III Instituto de Matematica e Estatistica, Universidade de Sao Paulo, Sao Paulo, SP, BR

**Keywords:** Hemodynamics, Human Comfort, Pregnant Women, Prone Position, Pregnancy

## Abstract

**OBJECTIVES:**

To analyze the influence of lying in prone position on a specially designed stretcher on the maternal-fetal hemodynamic parameters and comfort of pregnant women.

**METHODS:**

A randomized, controlled trial with 33 pregnant women divided into 2 groups: pregnant group sequence 1 and pregnant group sequence 2. The order of positions used in sequence 1 was Fowler’s position, prone position, supine position, left lateral, Fowler’s position 2, supine position 2, prone position 2 and left lateral 2. The order of positions used in sequence 2 was Fowler’s position, prone position, left lateral, supine position, Fowler’s position 2, left lateral 2, prone position 2 and supine position 2. Each woman remained in each position for 6 minutes. For the statistical analyses, we used Wilcoxon’s test for 2 paired samples when comparing the prone position with the other positions. The variables are presented in graphs showing the means and 95% confidence intervals. Trial Registration: Clinical Trial No. ISRCTN41359519

**RESULTS:**

All the parameters were within the standards of normality. There were no differences between positions in terms of maternal heart rate, diastolic blood pressure, oxygen saturation and fetal heart rate. However, there were significant decreases in respiratory rate and systolic blood pressure in prone position 2 compared with left lateral 2. There was an increase in oxygen saturation in prone position compared with Fowler’s position and supine position 2 in both sequences. All the women reported feeling comfortable in the prone position.

**CONCLUSIONS:**

The prone position was considered safe and comfortable and could be advantageous for improving oxygen saturation and reducing the systolic blood pressure and respiratory rate.

## INTRODUCTION

Maternal hemodynamics, in response to different body positions assumed during doctor appointments, clinical exams or physiotherapy treatment sessions, can influence fetal heart rate patterns 1. Aortocaval compression occurs when the maternal position causes the pregnant uterus to exert backwards force, thus causing a reduction in venous return, cardiac output and arterial blood pressure 2,3.

The maternal and fetal physiological changes in response to the maternal position have been studied by some authors 4-11 because they are of the utmost importance for selecting the best maternal position to avoid maternal and fetal intercurrences. In addition to hemodynamic changes, pregnant women can gain an additional 20% of their body weight and 100% mechanical overload on their joints 3. The pregnant uterus shifts the center of gravity forward, which can also cause tension and pain in the lumbosacral spine. These changes, together with hormonal changes, can cause painful syndromes, such as back pain or pregnancy-related posterior pelvic pain.

However, in the consulted literature, there were no studies of the physiological maternal-fetal hemodynamic parameters when pregnant women are placed on stretchers specifically designed for use in the prone position (PP) – a very important position for treating pain, back discomfort and acute respiratory distress syndrome – because it is difficult for pregnant women to maintain this position due to the abdominal volume during the gestation period. Bearing this in mind, this study developed and produced a prototype of a special stretcher to better accommodate pregnant women in different positions, and it aimed to verify the influence of the PP on the comfort and safety of both the mother and the fetus during pregnancy. Hence, the objectives of this study were to analyze the influence of the PP on maternal-fetal hemodynamic parameters and the comfort of pregnant women when lying on the prototype stretcher.

## MATERIALS AND METHODS

This randomized, controlled trial was conducted at the Department of Obstetrics at the Hospital das Clínicas da Faculdade de Medicina da Universidade de São Paulo (FMUSP) between April 2012 and March 2013 after the local ethics committee approved this study under number 0843/11. All the women signed written consent forms.

On the day of a routine prenatal check-up, each woman was referred to an interviewer. The pregnant women who were available to participate in the study on the day of their visits were asked to lie on the prototype stretcher in the designated positions after they were selected for the study, having matched the inclusion criteria and signed an informed consent form. The women who were not available for the study were scheduled for their next check-up.

The inclusion criteria were as follows: pregnant women between the ages of 20 and 34 years old with singleton uncomplicated pregnancies, alive fetus, a gestational age between 20 and 37 weeks, and no evidence of spinal disease. We included 33 healthy pregnant women, i.e., those without any pre-existing diseases, such as diabetes, chronic hypertension, or auto-immune, cardiovascular or renal diseases. Three pregnant women were in eligible because they missed their appointments. If the women reported any pain or discomfort on the prototype stretcher, they were excluded.

### Special stretcher for pregnant women

The main objective of physical therapy is to reduce or prevent pain, especially in the lumbar and dorsal regions. Bearing in mind the difficulty pregnant women have with feeling comfortable in the PP while undergoing physiotherapy for common joint pain in the spine, we identified the need for a specially designed stretcher. Therefore, we created one, and for 3 years, the stretcher underwent several adjustments in the Engineering Department of the University Santa Cecilia under the supervision of Prof. Carlos Alberto Amaral Moino, who is in charge of the integration of physical therapy and mechanical engineering. During this time, pregnant women were asked about possible improvements to the stretcher. After many adjustments, we chose the third prototype of the stretcher for this study. Because the impacts of the PP on the circulatory system of the mother and the fetus was one of our concerns, we decided to analyze the maternal and fetal parameters in the PP. The prototype stretcher that was used enabled the participants to remain in the PP without pressure on the abdominal region.

**Figure 1:**
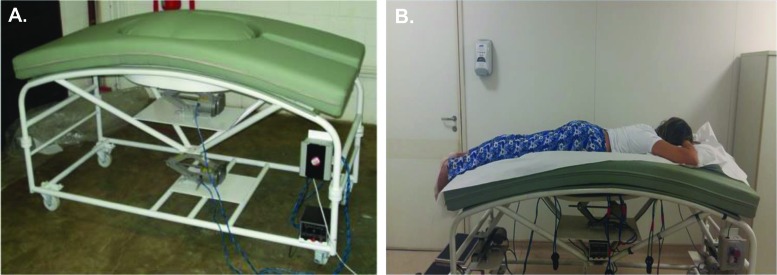
**A.** Lateral view of the prototype of the stretcher that was specifically designed for pregnant women. National Industrial Intellectual Property Institute (INPI Brazil) – BR 10 2014 017147 9 and International - Patent Cooperation Treaty (PCT/BR2015/000103) **B.** A pregnant woman in the prone position on the stretcherwith cervical support.

**Figure 2:**
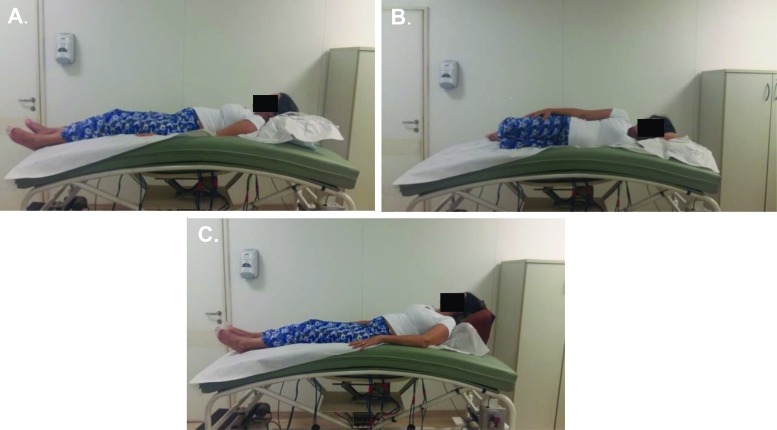
**A.** Supine position with a 15-degree tilt **B.** Left lateral position with support for the lower limbs **C.** Fowler’s position, i.e., semi-upright with slightly flexed knees and a 45° support placed behind the back.

This prototype stretcher was also created to enable pregnant women to remain not only in the PP but also in other positions that are useful in medical care (e.g., when conducting tests, such as ultrasounds) and in the hospital, such as positions for prescribed bed rest and those that facilitate surgical procedures on the spine or there moval of tumors. The stretcher height can be adjusted, and the stretcher has a round opening with a convex shape covered with a flexible foam pad of proper density that adapts to and comfortably accommodates the pregnant abdomen while the woman is lying in the PP (Figure 1). It also enables women to remain in the supine position (SP) with a 15-degree tilt; in the left lateral (LL) position with the lower limbs supported; and in Fowler’s position (FP), i.e., semi-upright with the knees slightly flexed and with a 45° support placed behind their backs (Figure 2).

This stretcher was registered with the National Industrial Intellectual Property Institute (INPI Brazil) – BR 10 2014 017147 9 and International - Patent Cooperation Treaty (PCT/BR2015/000103). A detailed description of the stretcher can be found at https://gru.inpi.gov.br/pePI/servlet/PatenteServletController.

### Demographic and clinical data

We collected the participants’ sociodemographic and clinical data (ethnicity, age, weight, height, smoking history, comfort, and gestational age and parity) and then classified them using the Atalah curve 12 as underweight, eutrophic, overweight or obese according to their body mass index (BMI) values.

After the sociodemographic data were collected, the women were asked to lie on the prototype stretcher for six minutes in each of the following positions: the SP with a 15-degree tilt; the LL position with support for the lower limbs; the PP with cervical support on the stretcher; and FP, i.e., semi-upright with slightly flexed knees and a 45° support placed behind the back.

The participants were divided into two groups –pregnant group sequence one (PGS1) and pregnant group sequence two (PGS2) – to assess the influence of the previous position on the maternal and fetal data during PP.

**Figure 3:**
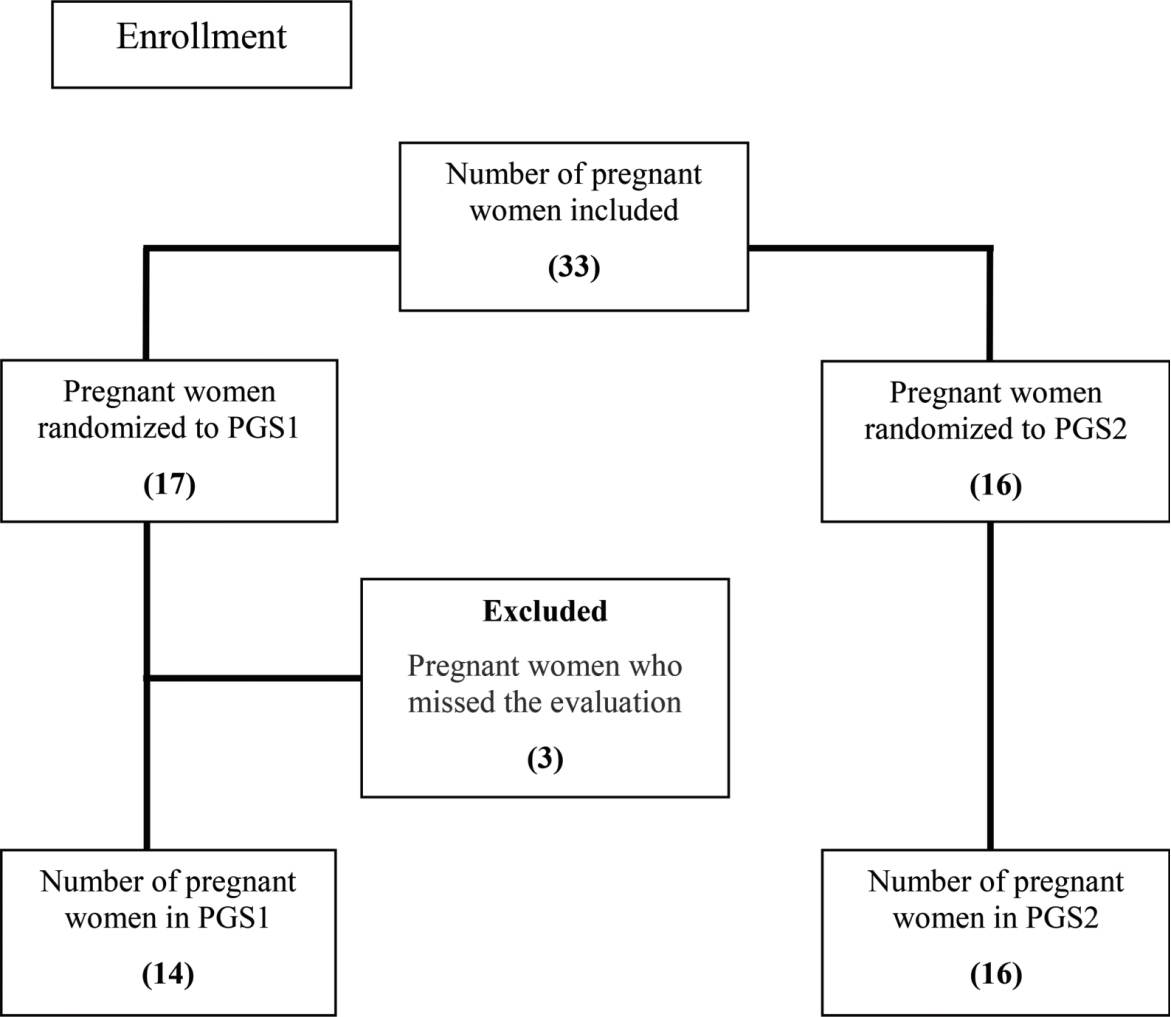
Flow chart of the total number of participants in pregnant group sequence 1 (PGS1) and pregnant group sequence 2 (PGS2) according to the order of randomization.

The sequence of positions for PGS1 was FP, PP, SP, LL, FP2, SP2, PP2 and LL2; for PGS2, the sequence was FP, PP, LL, SP, FP2, LL2, PP2 and SP2. The women were randomized to either PGS1 or PGS2 (the randomization list was created on 3 April 2012 using the Web site www.randomization.com; Figure 3).

The order of the positions in these two sequences was changed eight times to determine whether the order produced any significant variations in heart rate (HR), oxygen saturation (SpO2), systolic blood pressure (SBP), diastolic blood pressure (DBP) and respiratory rate (RR). These five maternal hemodynamic indices were measured using a Dixtal multiparameter monitor, model DX-2020(Campinas, São Paulo, Brazil). The standards of normality were HR at rest between 60 and 100 bpm; blood pressure up to 140/90 mmHg; RR between 16 and 22 rpm; and SpO2 between 95% and 100% 14. The fetal HR was between 110 and 160 bpm 7.

The methodology was standardized, and the tension levels were measured at the left upper arm. The cuff was placed 2.5 cm above the antecubital space. The RR was timed over1 minute of the respiratory cycle (inspiration and expiration).

To assess the fetal HR, cardiotocography was performed (Bistos n 049, Ribeirão Preto, São Paulo, Brazil). Each participant was asked to lie on the prototype stretcher in a calm environment with a mild temperature and to remain in the FP position for 10 minutes to stabilize the hemodynamic parameters prior to the first six-minute period of the sequence into which she had been randomized. All the women and fetuses were monitored during this process. Before shifting to the next position, each participant answered the questions “Are you comfortable?” and “Have you felt any discomfort in this position?”. All assessments were performed by the same researcher.

### Sample size

The sample size was calculated based on a pilot study of 10 pregnant women who were not included in this study. We observed a mean of 5 mm Hg and a standard deviation of 6 mm Hg in their blood pressures as they positioned themselves according to the assigned sequence. When considering a significance level of 5% and a power analysis of 80%, the sample size was estimated to require at least eleven cases for each sequence.

### Statistical analysis

Quantitative variables are summarized using medians and minimum and maximum values, and qualitative variables are presented as the means of absolute and relative frequencies (%). The associations between sequences (PGS1 and PGS2) and qualitative variables were investigated using Fisher’s exact test, and comparisons between sequences were performed using the Mann-Whitney test for demographic, quantitative variables.

The behaviors of the variables in each sequence are presented in graphs showing the means and 95% confidence interval (CI).

Wilcoxon’s test for two paired samples was used to compare the PP with the other positions. A p-value less than 0.05 was considered statistically significant. The analyses were performed using IBM SPSS software version 20.0 for Windows.

## RESULTS

The sociodemographic and clinical characteristics of the pregnant women in PGS1 and PGS2 are presented in Table 1. No differences were observed in the demographic or clinical characteristics between groups. Regardless of the position adopted, all clinical parameters were within the standards of normality.

**Figure 4:**
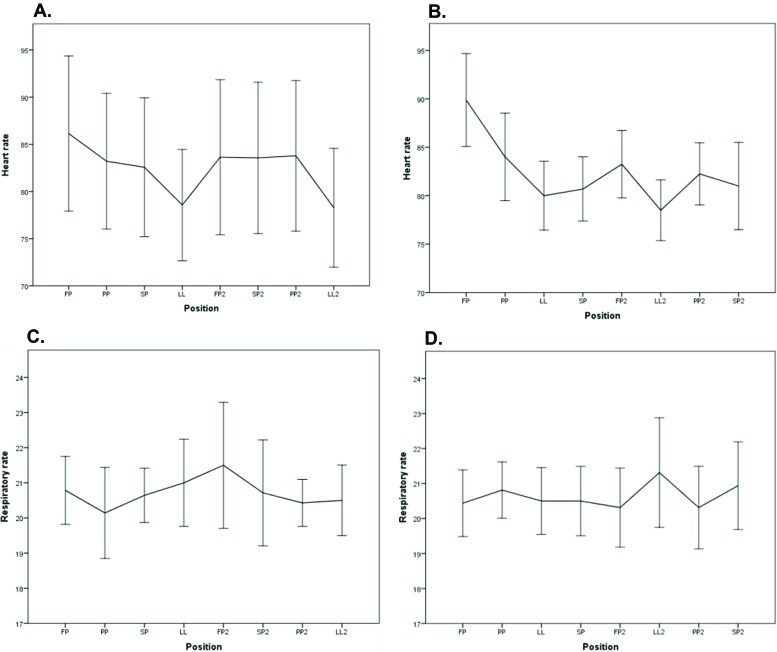
Graphical representation of the heart rates of the pregnant women who performed sequence 1 (A) and sequence 2 (B). Graphical representation of the respiratory rates of the pregnant women who performed sequence 1 (C) and sequence 2 (D).

There were no significant differences in HR when the women in PGS1 shifted from FP to PP (*p*=0.220) or from SP2 to PP2 (*p*=0.844). There were significant differences in HR when the women in PGS2 shifted from FP to PP (*p*=0.012), but no significant difference occurred when they shifted from LL2 to PP2 (*p*=0.070; Figure 4).

There was no significant difference in RR when the women in PGS1 shifted from FP to PP (*p*=0.255) or from SP2 to PP2 (*p*=0.959). In PGS2, there was no significant difference in RR when the women shifted from FP to PP (*p*=0.319), but there was a significant difference when they shifted from LL2 to PP2 (*p*=0.031; Figure 4).

**Figure 5:**
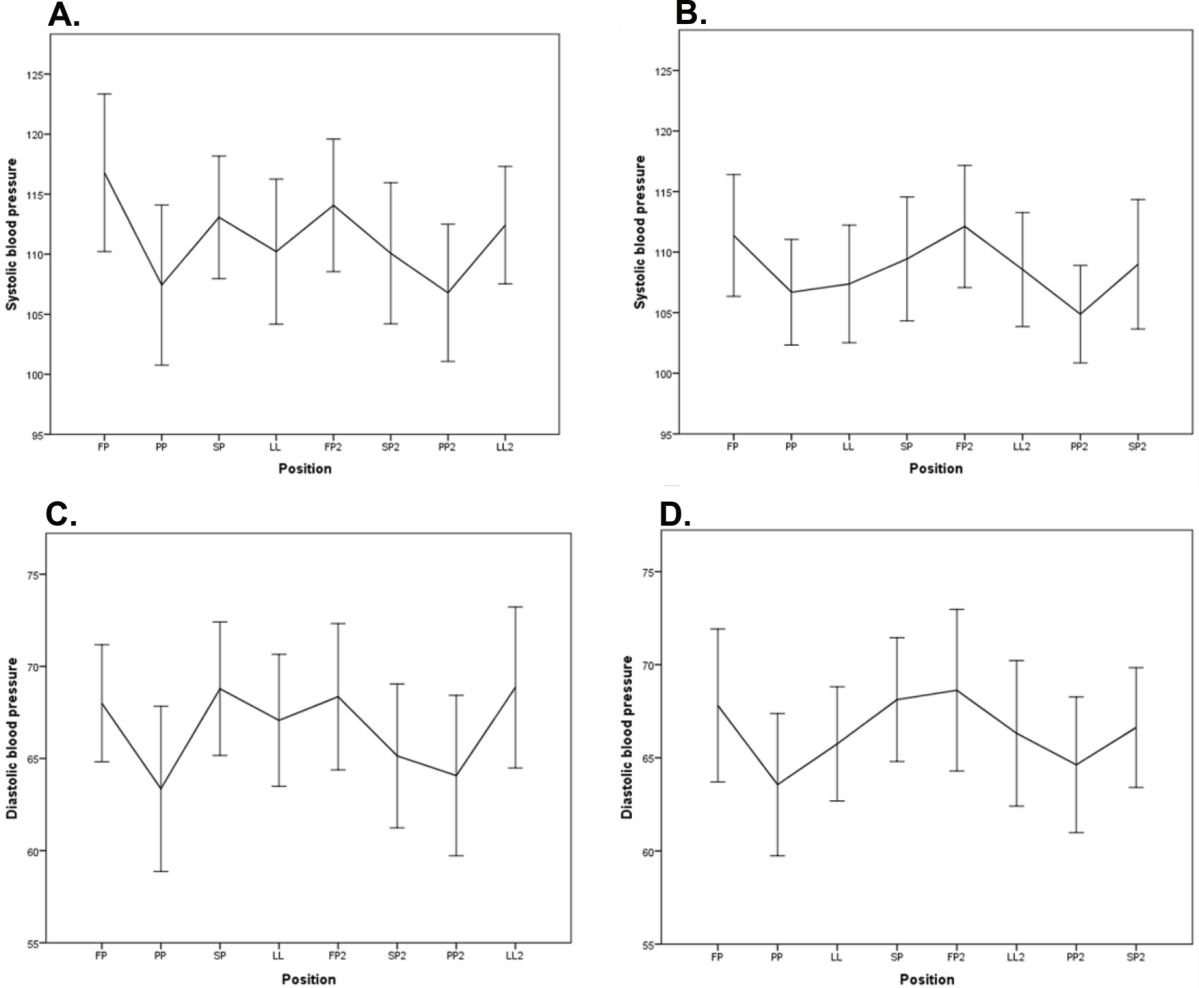
Graphical representation of the systolic blood pressure of the pregnant women who performed sequence 1 (A) and sequence 2 (B). Graphical representation of the diastolic blood pressure of the pregnant women who performed sequence 1 (C) and sequence 2 (D).

There was a significant difference in SBP between FP and PP (PGS1: *p*=0.005 and PGS2: *p*=0.005). In PGS1, there were no significant differences when the women shifted from SP2 to PP2 (*p*=0.142), but in PGS2, there was a significant difference when they shifted from LL2 to PP2 (*p*=0.026; Figure 5).

There was a significant difference in DBP between FP and PP for both groups (PGS1: *p*=0.025 and PGS2: *p*=0.028), but there were no significant differences when the women in PGS1 shifted from SP2 to PP2 (*p*=0.599) or when they shifted from LL2 to PP2 (S2: *p*=0.245; Figure 5).

**Figure 6:**
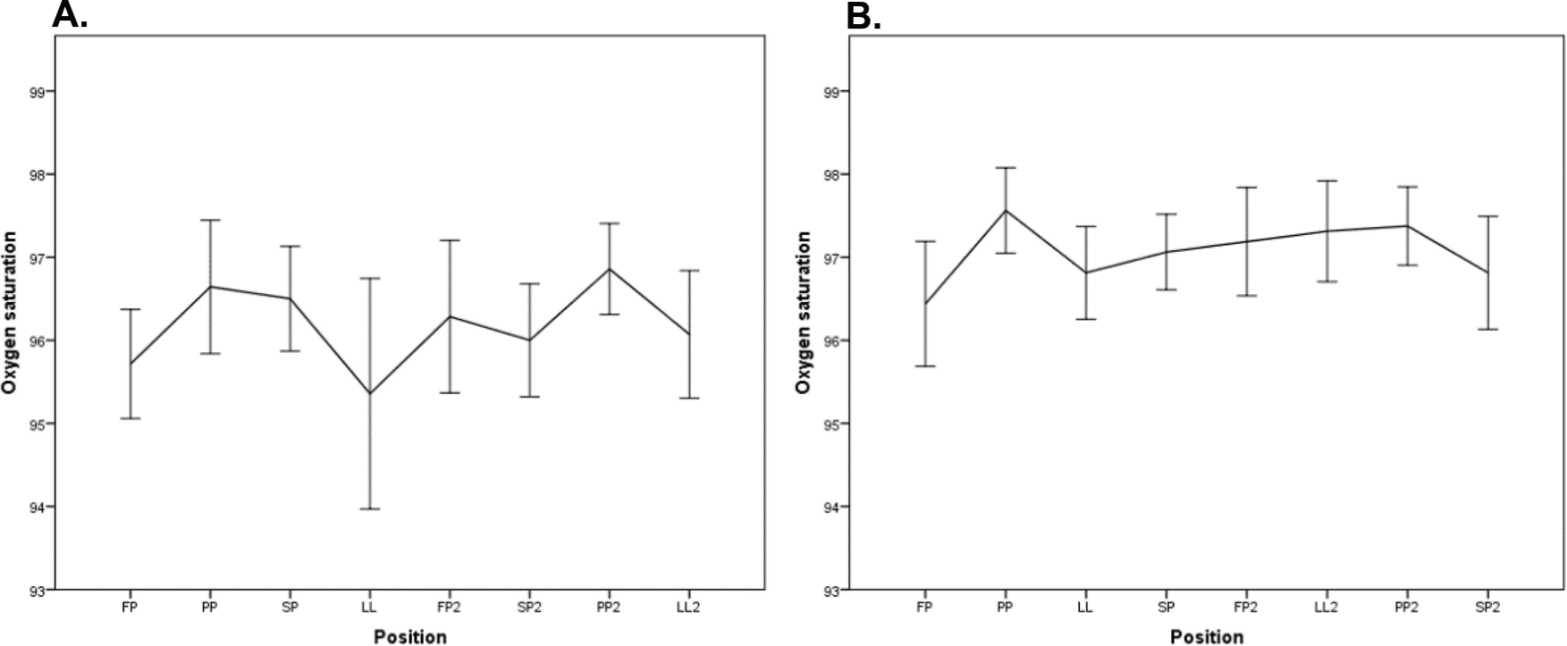
Graphical representation of the oxygen saturation of the pregnant women who performed sequence 1 (A) and sequence 2 (B).

There was a significant difference in SpO_2_ between FP and PP for both groups (PGS1: *p*=0.036 and PGS2: *p*=0.008). In PGS1, there was also a significant difference when the women shifted from SP2 to PP2 (*p*=0.010), but there was no significant difference when the women in PGS2 changed from LL2 to PP2 (*p*=0.822; Figure 6).

**Figure 7:**
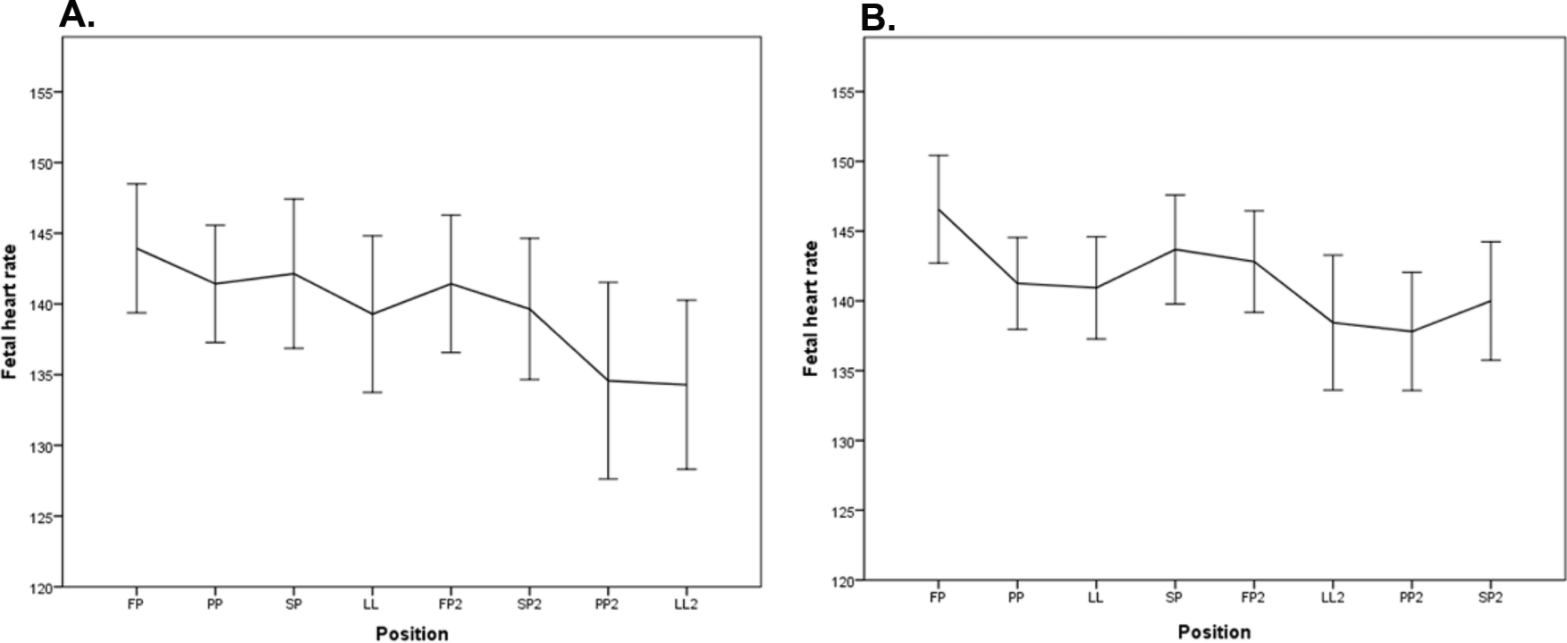
Representation of the fetal heart rates of the fetuses of the pregnant women who performed sequence 1 (A) and sequence 2 (B).

There was no significant difference in baseline fetal HR between FP and PP in PGS1 (*p*=0.070), but there was a difference in PGS2 (*p*=0.013).There was a significant difference in baseline fetal HR between SP2 and PP2 in PGS1 (*p*=0.031) but no significant difference when LL2 was compared with PP2 in PGS2 (*p*=0.608; Figure 7).

All the women responded positively when asked if they were feeling comfortable on the prototype stretcher during the two sequences of positions.

## DISCUSSION

According to the results presented here, when the participants were placed on the prototype stretcher in the PP, maternal HR, SBP, DBP, RR, SpO_2_, and baseline fetal HR were within normal limits, independent of the position adopted. All the women felt comfortable in all positions.

When a pregnant woman is positioned on a traditional stretcher, some factors must be considered. First, the chosen position can influence the maternal-fetal hemodynamic parameters, mainly after the 20th gestational week 14. Second, the selected position can cause discomfort for women whose abdomens are larger due to pregnancy.

These factors led us to build a prototype stretcher that allows pregnant women to remain in a number of varied positions comfortably and without changes to maternal or fetal hemodynamics. We tested this specially designed stretcher and found that all parameters remained within the limits of normality, even though all the study participants were at more than 20 weeks gestation.

There was a significant difference in maternal HR between FP and PP in sequence 2 but not in sequence 1. This difference could be explained by the fact that the mean HR in sequence 2 was greater than that in sequence 1, and it is possible that this higher mean HR increased the effect of the change in position. The same outcome was observed for fetal HR. Despite this finding, it is important to consider that we observed lower values of maternal and fetal HR in the PP than in FP in the two sequences. In this study, we observed a significant decrease in the RR from LL2 to PP2, a decrease in systolic blood pressure from FP to PP and from LL2 to PP2 in both groups, a decrease in diastolic blood pressure from FP to PP in both groups and increases in SpO_2_ from FP to PP and from SP2 to PP.

Several studies have indicated that the LL position is the best position for a pregnant woman on a traditional stretcher 5,8,9,15,16,17. However, prior to this study, there were no references in the medical literature to the PP on stretchers specifically designed for pregnant women.

There were no differences in HR, DBP, SpO_2_ or fetal HR when the LL and PP positions were compared. However, there was a significant a decrease in RR and SBP.

Based on these results, the PP can be presented as an alternative to the LL position, and it might even be advantageous in situations in which there is a benefit to reducing SBP and RR.

An increase in SpO_2_ in the PP and the SP compared with sitting was also found. These findings represent an advance because they provide pregnant women with a different way to improve oxygenation.

Two reports of pregnant women in the PP were published. One of the pregnant women suffered from acute respiratory distress syndrome 18, and the other woman required surgery for cauda equina syndrome and obesity 19. The use of the PP for these women was associated with an improvement in SpO_2_. Pillow rolls were used as supports for the chest region and the ilium bone. According to these studies, the use of the PP was limited because until that date, there was no stretcher specifically designed for pregnant women.

There are two published case reports of pregnant women in the PP. One case was a pregnant woman who was scheduled to undergo surgery on a lumbar disc at week 27 of her pregnancy 20, and the other was a pregnant woman who was scheduled to undergo posterior fossa craniotomy at week 20 21.

This study was the first to investigate maternal-fetal hemodynamic abnormalities in pregnant women placed in the PP for at least 6 minutes on a specially designed stretcher. Because there were no abnormalities, this position proved to be safe when assumed on the prototype stretcher. Therefore, the PP can be used by pregnant women when they undergo examinations, anesthesia or physiotherapeutic procedures.

In addition to studying hemodynamic variations, and because pregnancy generates an overload on the posterior muscle chain that can cause women to experience discomfort and pain, we also assessed the comfort of the women while they were on the prototype stretcher 22. The SP is considered the most uncomfortable position by 64.7% of pregnant women 23. Nevertheless, while lying on the prototype stretcher, the participants in this study reported that they were completely comfortable in all positions, including the SP and the PP, which are traditionally considered uncomfortable on conventional stretchers. This result confirmed the distinctive characteristics of the stretcher used in this study. The hole in the center of the stretcher ensures a perfect fit for the pelvic area, and it is covered with material that has a specific density. In addition, the prototype stretcher includes supports with different densities for preventing lumbar lordosis. Conventional stretchers do not have these features.

All the assessed women indicated that they were comfortable and experienced a feeling of well-being while they were in the PP on the prototype stretcher. According to statements provided by several participants, the PP was their favorite position before and during pregnancy. This positive feedback was due to the prototype stretcher’s convex shape, which adapted to the respiratory diaphragm and allowed free diaphragm extension, which improves oxygenation. The shape promotes physical relaxation, which is important because it reduces maternal anxiety and can have a direct and positive effect on fetal HR patterns 24. All the fetuses in this study maintained baseline fetal HRs with in normal parameters in all the positions the pregnant women assumed on the prototype stretcher.

The limitations of this study are the same as those of other studies. This study did not evaluate the PP during different gestational trimesters, given the variation in physiological parameters according to gestational age, and the women were limited to 6 minutes in each position. Another limitation of this study is that the PP was evaluated at different stages of pregnancy, between 20 and 37 weeks of gestation. Given the variation in physiological parameters according to gestational age, it could be interesting to analyze the specific changes at each week of pregnancy. A third limitation was the length of time spent in each position, which was limited to 6 minutes. Considering the possibility of using the prototype stretchers in surgery, further studies are needed to evaluate the results of longer periods in the PP. The main limitation was that we could not compare our results with those of other studies because there were no reports of stretchers similar to the one used in this study.

Hence, this is a starting point for new studies that can investigate whether the PP is the best position for various procedures, such as analyzing pulmonary ventilation in pregnant women with lung diseases, analyzing blood pressure in pregnant women who suffer from hypertension, and analyzing back diseases or other diseases that require the patient to lie in the PP for treatment.

In conclusion, the present study showed that with the use of a prototype stretcher designed for pregnant women, the PP was considered a safe, comfortable position and that it could even be advantageous in situations in which there is a benefit to reducing SBP and RR and improving SpO2.

## AUTHOR CONTRIBUTIONS

Oliveira C designed the study, analyzed the data, wrote the manuscript, reviewed the analysis of the data and approved the final version of the manuscript. Lopes MA reviewed the analysis of the data and approved the final version of the manuscript. Rodrigues AS reviewed the statistical analysis. Zugaib M reviewed the analysis of the data and helped to conduct the study. Francisco RP helped to design the study, analyzed the data and helped to write the manuscript. All the authors read and approved the manuscript.

## Figures and Tables

**Table 1:** Sociodemographic and clinical characteristics of the study participants in PGS1 and PGS2.

Characteristics		PGS1 (n=14)	PGS2 (n=16)	*p*-value
Ethnicity, n (%)	White	9 (64.3)	11(68.8)	0.999^a^
	Non-white	5 (35.7)	5 (31.3)	
Smoking, n (%)	Yes	0 (0)	0 (0)	-
	No	14 (100)	16 (100)	
Parity, n (%)	0	6 (42.9)	11 (68.8)	0.241^b^
	1	4 (28.6)	4 (25)	
	2	2 (7.1)	1 (6.3)	
	3 or more	3 (21.4)	0	
Median maternal age (range)	-	28.50 (20-34)	27.63 (20-34)	0.552^b^
Median body mass index (range)	**-**	24.82 (19-30)	22.61 (16-31)	0.093^b^

a Fisher’s exact test;

b Mann-Whitney test
